# The value of conventional ultrasound combined with superb microvascular imaging and color Doppler flow imaging in the diagnosis of thyroid malignant nodules: a systematic review and meta-analysis

**DOI:** 10.3389/fendo.2023.1182259

**Published:** 2023-06-21

**Authors:** Li Jiang, Dian Zhang, Yue-Nan Chen, Xue-Juan Yu, Mei-Fang Pan, Lian Lian

**Affiliations:** ^1^ Medical College, Yangzhou University, Yangzhou, Jiangsu, China; ^2^ Department of Ultrasound, Suzhou Xiangcheng People’s Hospital, Jiangsu, China; ^3^ Department of Oncology, Suzhou Xiangcheng People’s Hospital, Jiangsu, China

**Keywords:** malignant, thyroid nodules, ultrasonography, Superb microvascular imaging, color Doppler flow imaging, meta-analysis

## Abstract

**Purpose:**

To evaluate and compare the value of conventional ultrasound-based superb microvascular imaging (SMI) and color Doppler flow imaging (CDFI) in the diagnosis of malignant thyroid nodule by meta-analysis.

**Methods:**

The literature included in the Cochrane Library, PubMed, and Embase were searched by using “ superb microvascular imaging (SMI), color Doppler flow imaging (CDFI), ultrasound, thyroid nodules” as the keywords from inception through February 1, 2023. According to the inclusion and exclusion criteria, the clinical studies using SMI and CDFI to diagnose thyroid nodules were selected, and histopathology of thyroid nodules was used as reference standard. The diagnostic accuracy research quality assessment tool (QUADAS-2) was used to evaluate the quality of included literature, and the Review Manager 5.4 was used to make the quality evaluation chart. The heterogeneity test was performed on the literature that met the requirements, the combined sensitivity, specificity, positive likelihood ratio, and negative likelihood ratio were pooled, and a comprehensive ROC curve analysis was performed. Meta-DiSc version 1.4, StataSE 12, and Review Manager 5.4 software were used.

**Results:**

Finally, 13 studies were included in this meta-analysis. A total of 815 thyroid malignant nodules were assessed. All thyroid nodules were histologically confirmed after SMI or CDFI. The combined sensitivity, specificity, PLR, NLR, DOR, and area under the SROC curve of SMI for the diagnosis of malignant thyroid nodules were 0.80(95%CI: 0.77-0.83), 0.79(95%CI: 0.77-0.82), 4.37(95%CI: 3.0-6.36), 0.23(95%CI: 0.15-0.35), 22.29(95%CI: 12.18-40.78), and 0.8944, respectively; the corresponding values of CDFI were 0.62(95%CI: 0.57-0.67), 0.81(95%CI: 0.78-0.85), 3.33(95%CI: 2.18-5.07), 0.41(95%CI: 0.27-0.64), 8.93(95%CI: 3.96-20.16), and 0.8498. Deek funnel pattern showed no significant publication bias.

**Conclusion:**

The diagnostic efficiency of SMI for malignant thyroid nodules is better than CDFI, and SMI technology can provide significantly more information on vascularity, make up for the deficiency of CDFI, and has better clinical application value.

**Systematic review registration:**

https://www.crd.york.ac.uk/PROSPERO, identifier CRD42023402064.

## 1 Introduction

In recent years, the incidence rate of thyroid cancer is on the rise. With the wide application of ultrasound technology, ultrasound has become one of the most important means of thyroid detection (1, 2). Fine needle aspiration biopsy (FNA) is the best non-surgical diagnostic method for thyroid malignant tumor at present, but it is an invasive examination (3–6). Improving the accuracy of non-invasive ultrasound diagnosis is helpful to reduce the number of FNA, which is of clinical significance. Although ultrasound has made significant progress in the differential diagnosis of benign and malignant thyroid nodules, at present, conventional ultrasound (CUS) combined with color Doppler flow imaging (CDFI) still has a certain rate of missed diagnosis and misdiagnosis in determining the nature of thyroid nodules, and the diagnostic accuracy is not high, and there are also technical limitations in detecting small blood vessels and low blood flow (7, 8). As a new vascular imaging technology, Superb microvascular imaging (SMI) can visualize low-velocity blood flow (9, 10). Compared with CDFI, it can describe blood flow in more detail, and can obtain high-quality microvascular images without relying on contrast media, it can also describe the blood flow around the sinus and in the nodule in more detail (11, 12). In recent years, with the wide application of SMI technology in breast, liver, and other fields, more and more research teams have begun to pay attention to its diagnostic value for thyroid nodules (13–18). Although there are many related studies comparing SMI with CDFI, there are still disputes on the comparison of diagnostic indicators such as sensitivity and specificity. Therefore, this study included relevant literature and applied Meta-analysis to comprehensively compare the diagnostic efficacy of the two methods in order to provide a more objective and comprehensive evidence-based basis for the diagnosis and clinical management of thyroid nodule disease.

## 2 Materials and methods

### 2.1 Search strategy

We searched PubMed, Embase, and Cochrane Library from inception through February 1, 2023. The following keywords and MeSH terms were used: [“thyroid cancer” or “thyroid tumor” or “thyroid nodule”] and [“superb microvascular imaging” or “SMI”] and [“color Doppler flow imaging” or “CDFI”] and [“ultrasound”]. We also performed a manual search to find other potential articles. Two investigators (LJ and DZ) searched online to obtain the original data, and the reference lists of all relevant articles were also scanned. All retrieved citations were exported to Zotero and checked for duplicates.

### 2.2 Inclusion and exclusion criteria

The inclusion criteria of this study were as follows:(1) Clinical cohort study or diagnostic test; (2) All thyroid nodules were histologically confirmed after ultrasonography; (3) All studies can directly or indirectly obtain original data such as true positive (TP), false positive (FP), false negative (FN), true negative values (TN). Exclusion criteria: (1) Abstracts, reviews, or case reports; (2) Repeated publication of data; (3) Incomplete original data.

### 2.3 Data extraction

Two evaluators (LJ and DZ) independently screened the literature according to the inclusion and exclusion criteria of the literature, and finally obtained two copies of data, and then cross-checked the data. If there were different opinions, discuss and negotiate together or ask a third party to help decide. Extract literature data from the data, including the first author, publication year, country, number of malignant lesions, the average age of patients, instrument system parameters, diagnostic gold standard, and research type. This study was conducted by the Preferred Reporting Items for Systematic Reviews and Meta-Analyses (PRISMA) guidelines (19), the protocol was registered in the PROSPERO:(CRD42023402064).

### 2.4 Literature quality assessment

The diagnostic accuracy research quality evaluation tool (QUADAS-2) recommended by Cochrane was used to evaluate the quality of the included literature, and 14 items such as the population to be evaluated in the diagnostic test and the gold standard were scored (20). According to the specific content of each item, the answer options are divided into “Yes”, “No” and “Unclear”, and then the risk of bias and clinical applicability is comprehensively evaluated. Revman 5.4 software is used to make quality evaluation charts.

### 2.5 Statistical analysis

Meta-DiSc version 1.4 (Universidad Complutense, Madrid, Spain) software was used for meta-analysis. The sensitivity (Sen), specificity (Spe), positive likelihood ratio (LR+), and negative likelihood ratio (LR-) are calculated, and the threshold effect is evaluated using the 95% confidence interval (CI). The summary receiver operating characteristic (SROC) curve and the corresponding area under the curve was determined. Quality evaluation chart was created using Review Manager (RevMan, version 5.4, Cochrane IMS). The heterogeneity of the diagnostic odds ratio (DOR) of each study was analyzed by Meta-Disc 1.4 software. If I^2^>50% or P<0.05 among the included literature, there is a high heterogeneity between the results; I^2^<25% indicates that the heterogeneity between results is small, and 25% ≤ I^2^ ≤ 50% indicates that the heterogeneity of results is medium; P ≥ 0.05 indicates that there is no heterogeneity in the results. If there is heterogeneity, try to explore the source of heterogeneity by using Meta-regression. The sensitivity analysis was carried out by StataSE 12 (Stata Corporation, College Station, TX). P<0.05 indicated a statistically significant difference.

## 3 Results

### 3.1 Overall characteristics of selected studies

**Figure 1:**
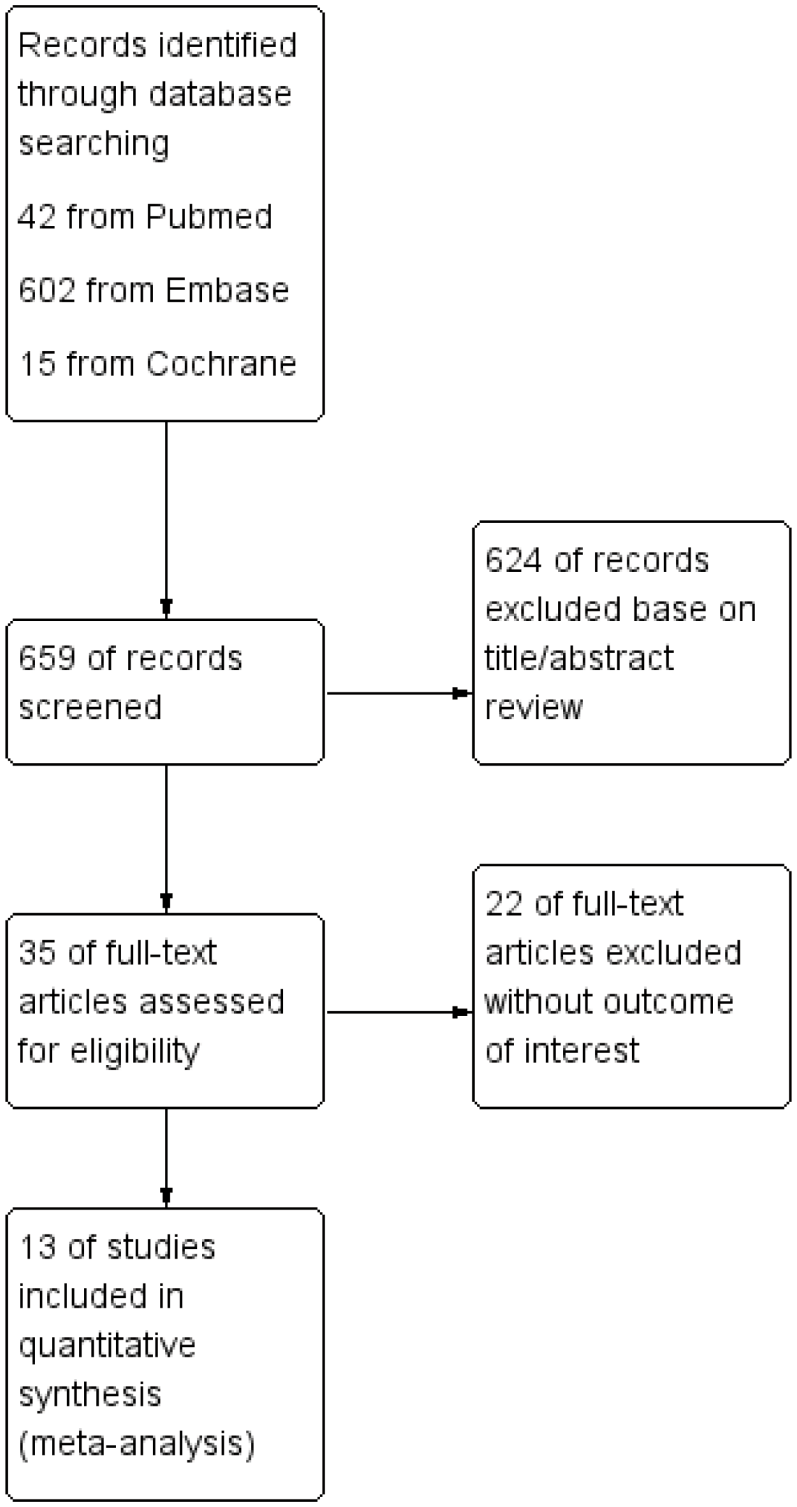
Flowchart of the study selection process.

After the primary election, the abstracts and titles of 659 published articles (42 from PubMed, 602 from Embase, and 15 from Cochrane) were preliminarily reviewed. After eliminating duplicate results and abstract screening, the number of complete publications that may meet the criteria was reviewed, and finally, 13 articles were included (21–33). Other 646 articles were excluded because of duplication, unrelated research, inappropriate data, review, no or inappropriate control group, other test methods or non-full text. **Figure 1** shows a flowchart that reflects the search process and the reasons of exclusion.

### 3.2 Characteristics of the included studies

The detailed characteristics of the included study are shown in **Table 1**, including the name of the first author, year of publication, the age range of patients, gender distribution (male/female), design, sample size, and equipment. All these articles were published from 2017 to 2022. The study was conducted in European countries (n=1), China (n=9), and Korea (n=3). The sample size is between 52 and 254. A total of 1712 nodules were included in these studies. After excluding invalid nodules, there were 897 benign nodules and 815 malignant nodules, respectively.

**Table 1:** Summary of included study characteristics.

Author (Year)	Country	Design	Sex (M/F)	Age in years (mean ± SD)	Nodules (n)	Malignant nodules (n)	SMI system
Zhu YC (2018) (21)	China	Prospective	35/41	49.62 ± 13.19	76	29	Toshiba Aplio500
Zhu YC (2021) (22)	China	Prospective	Not Available	57.89 ± 8.02	183	71	Toshiba Aplio 500
Yoon JH (2018) (23)	Korea	Prospective	31/138	50.3 ± 13	171	63	Toshiba Aplio 500
Xue SW (2021) (24)	China	Prospective	47/73	46.83 ± 8.20	120	50	Not Available
Pei S (2019) (25)	China	Retrospective	58/112	Not Available	196	118	Toshiba Aplio 500
Ahn HS (2017) (26)	Korea	Prospective	9/48	51.6 ± 11.2	52	26	Toshiba Aplio 500
Hong MJ (2022) (27)	Korea	Retrospective	10/42	51.2 ± 11.9	60	37	Toshiba Aplio 500
Kong J (2017) (28)	China	Retrospective	48/44	42	113	79	Toshiba Aplio 400
Yongfeng Z (2019) (29)	China	Prospective	14/62	43.09 ± 10.57	102	73	Toshiba Aplio 500
Zhang L (2020) (30)	China	Prospective	19/38	45.26 ± 12.30	75	40	Toshiba Aplio 500
Zhang L (2017) (31)	China	Retrospective	16/45	48.51 ± 15.50	68	28	Toshiba Aplio 500
Li Y (2017) (32)	China	Prospective	53/188	39 ± 16.5	254	73	Toshiba Aplio 500
Cappelli C (2019) (33)	Italy	Prospective	50/186	54 0 ± 12 5	246	128	Toshiba Aplio 500

### 3.3 Methodologic quality of the included studies

**Figure 2:**
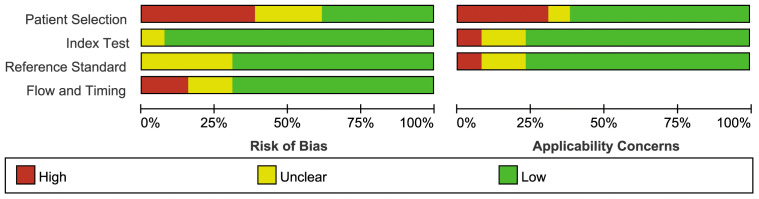
Percentage of included studies with the risk of bias.

**Figure 3:**
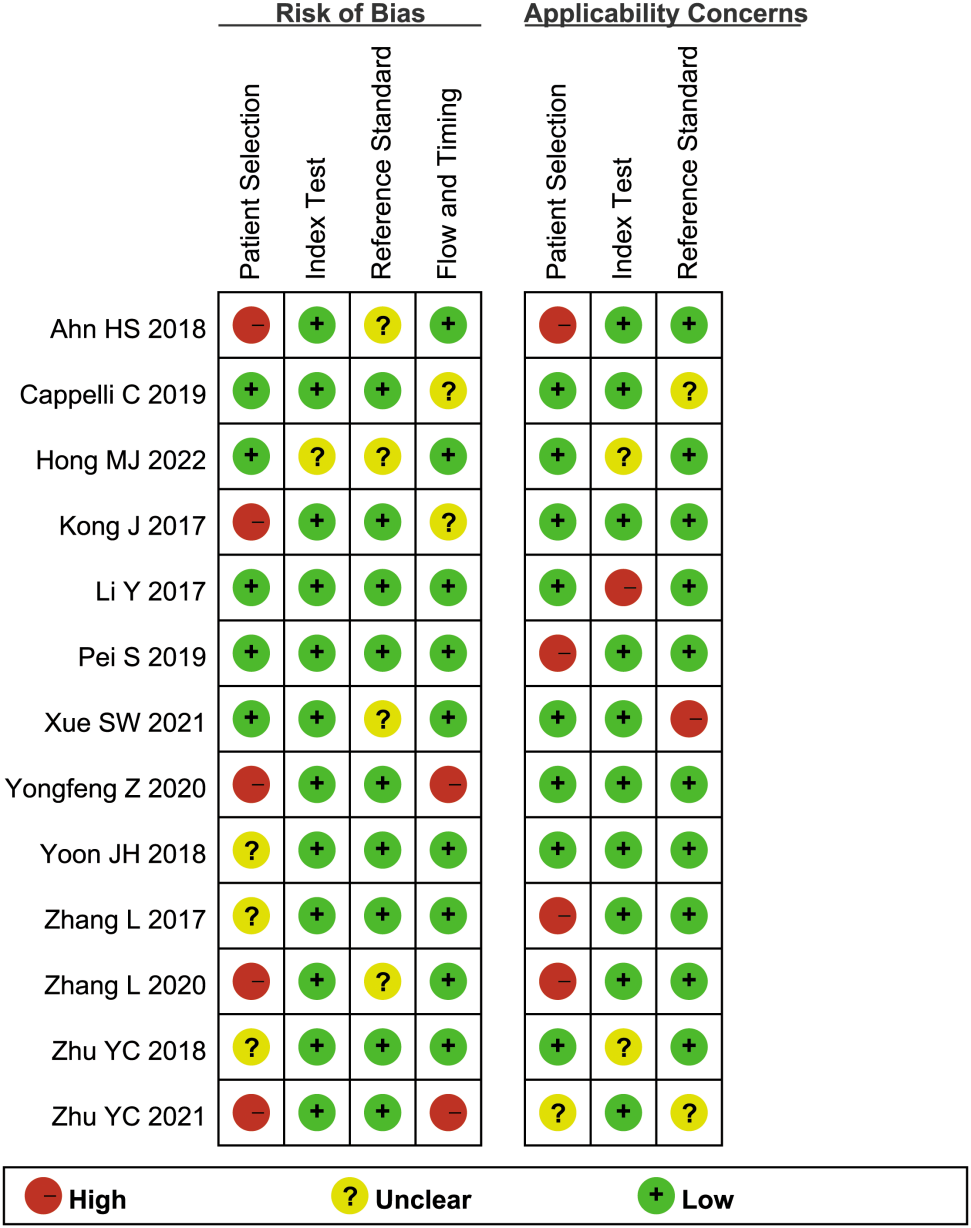
The assessment of the risk of bias for included study. Quality is represented by colors using green (+) as yes (high quality), yellow (?) as unclear, and red (–) as no (low quality).

**Figure 2** illustrates the methodological quality of the included studies based on QUADAS-2. **Figure 3** summarizes the risk of bias and adherence of individual studies to these items. **Table 2** provides data on the other items of methodological quality. All included studies had a low risk of bias and were of high quality.

**Table 2:** Summary of risk of bias for nine additional items assessing the methodologic quality of the included studies.

First Author [Reference No.]	Thresholds Established?	Technology Unchanged?	Positive Results Defined?	Appropriate Training?	Treatment Withheld?	Observer Variation Reported?	Instrument Variation Reported?	Objectives Prespecified?	Free of Commercial Funding?
Zhu YC (21)	Yes	Yes	Yes	Yes	Yes	Yes	Yes	Yes	Unclear
Zhu YC (22)	Yes	Yes	Yes	Yes	Yes	Yes	Yes	Yes	No
Yoon JH (23)	No	Yes	Yes	Yes	Yes	Unclear	Yes	Yes	Unclear
Xue SW (24)	Yes	Yes	Yes	Unclear	Unclear	Unclear	Yes	Yes	No
Pei S (25)	Yes	Yes	Yes	Unclear	Yes	Yes	Yes	Yes	Unclear
Ahn HS (26)	No	Yes	Yes	Unclear	Yes	Yes	Yes	Yes	Yes
Hong MJ (27)	No	Yes	Yes	Unclear	Yes	Yes	Yes	Yes	Yes
Kong J (28)	Yes	Yes	Yes	Unclear	Yes	Yes	Yes	Yes	Unclear
Yongfeng Z (29)	Yes	Yes	Yes	Yes	Unclear	Yes	Yes	Yes	Unclear
Zhang L (30)	Yes	Yes	Yes	Unclear	Yes	Yes	Yes	Yes	No
Zhang L (31)	Yes	Yes	Yes	Unclear	Yes	Yes	Yes	Yes	Unclear
Li Y (32)	Yes	Yes	Yes	Unclear	Unclear	Unclear	Yes	Yes	Unclear
Cappelli C (33)	No	Yes	Yes	Unclear	Yes	Yes	Yes	Yes	Unclear

### 3.4 Threshold effects and heterogeneity

The Spearman correlation coefficients were 0.429 and 0.512 by heterogeneity analysis (P>0.05), indicating that there was no threshold effect. At the same time, the results showed that heterogeneity for sensitivity (I^2 ^= 86.4%), specificity (I^2 ^= 88.0%) in SMI group and sensitivity (I^2 ^= 91.0%), specificity (I^2 ^= 66.5%) in CDFI group. The included literature has high heterogeneity, so it is necessary to use random effect model to summarize and evaluate and draw SROC curve.

### 3.5 Sensitivity analysis

**Figure 4:**
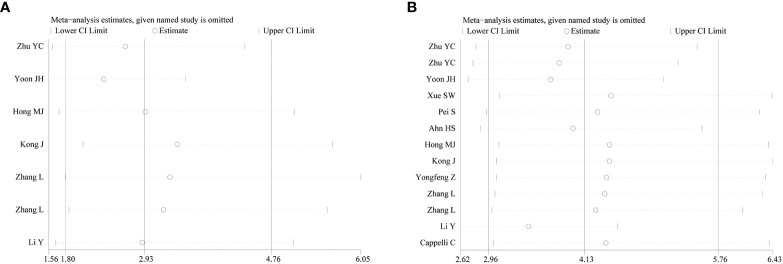
Sensitivity analysis of studies. [**(A)** CDFI; **(B)** SMI].

To observe the stability of the synthetic results, the data included in the literature were excluded one by one and the sensitivity and specificity were summarized again. It showed that the combined effect of various indicators changed little, indicating that the stability of the included literature was good and the reliability of the results was high (**Figure 4**).

### 3.6 Meta-regression analysis

As a result of the significant heterogeneity, meta-regression analysis was used to explore the source of heterogeneity. The covariates of the regression model are set as follows: (1) The sample size ≥ 100 is set as 1, and the sample size<100 is set as 0; (2) The age≥50 years old is set as 1, and the age<50 years old is set as 0; (3) The prospective study was set as 1, the retrospective study was set at 0; (4) The research object from China is set as 1, and that from other countries is set as 0; (5) The TI-RADS classification of nodules in the literature is not set as 1, and the TI-RADS classification of only 4 types of nodules in the literature is 0. Meta-regression analysis of the CDFI group and SMI group showed that there was no significant difference between the sources of heterogeneity and the covariates (P>0.05).

### 3.7 Diagnostic accuracy

**Figure 5:**
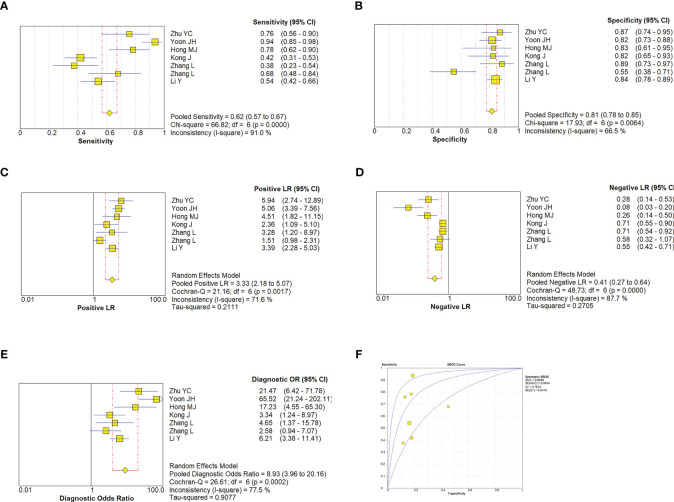
Estimates of CDFI assessment for the diagnosis of malignancy thyroid nodules. **(A–E)**, Forest plots illustrate pooled estimates (diamonds) for sensitivity **(A)**, specificity **(B)**, positive likelihood ratio (LR) **(C)**, negative LR **(D)**, and diagnostic odds ratio **(E) **and corresponding 95% CIs for pooled estimates. **(F)**, Summary receiver operating characteristic (SROC) plot for assessing accuracy with corresponding curves indicative of upper and lower bounds of 95% CI. AUC, area under curve; SE, standard error; Q* = summary measure of accuracy derived from the SROC curve.

**Figure 6:**
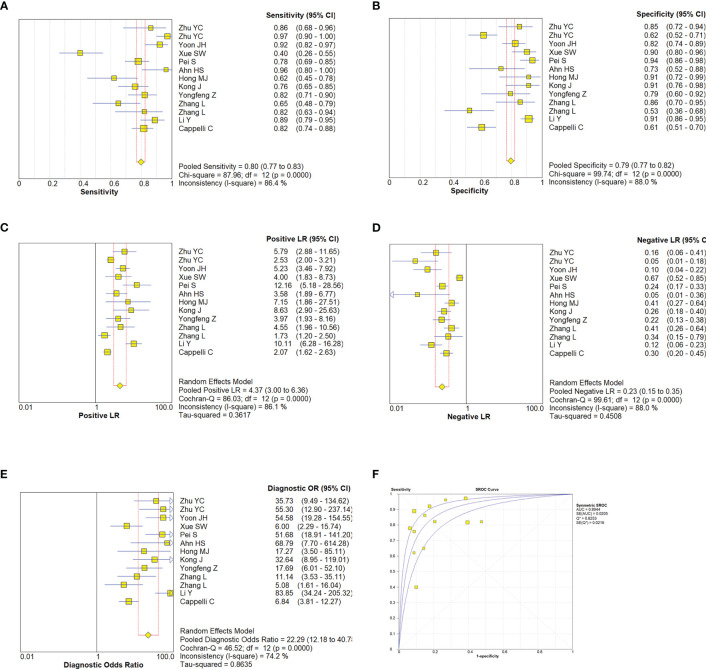
Estimates of SMI assessment for the diagnosis of malignancy thyroid nodules. **(A–E)**, Forest plots illustrate pooled estimates (diamonds) for sensitivity **(A)**, specificity **(B)**, positive likelihood ratio (LR) **(C)**, negative LR **(D)**, and diagnostic odds ratio **(E) **and corresponding 95% CIs for pooled estimates. **(F)**, Summary receiver operating characteristic (SROC) plot for assessing accuracy with corresponding curves indicative of upper and lower bounds of 95% CI. AUC, area under curve; SE, standard error; Q* = summary measure of accuracy derived from the SROC curve.

A random effect model was used to analyze the combined effect quantity of the diagnostic four-grid data of CDFI and SMI included in the literature. The combined sensitivity of CDFI and SMI in the diagnosis of thyroid nodules was 0.62(95% CI: 0.57-0.67) and 0.80(95% CI: 0.77-0.83), respectively; The combined specificity was 0.81(95% CI: 0.78-0.85) and 0.79(95% CI: 0.77-0.82) respectively; The positive likelihood ratios were 3.33(95% CI: 2.18~5.07) and 4.37(95% CI: 3.0~6.36) respectively; The negative likelihood ratios were 0.41(95% CI: 0.27~0.64) and 0.23(95% CI: 0.15~0.35) respectively; The DOR was 8.93(95% CI: 3.96~20.16) and 22.29(95% CI: 12.18~40.78) respectively. The area under the SROC curve is 0.8498 and 0.8944 respectively. The diagnostic sensitivity, positive likelihood ratio, and DOR of SMI were higher than those of CDFI, while the negative likelihood ratio was lower than that of CDFI. The SROC curve of SMI diagnosis is closer to the upper left corner of the image, and its area under the SROC curve is larger, indicating that the diagnosis efficiency of SMI is better than that of CDFI (**Figures 5**, **6**).

### 3.8 Evaluation of publication bias and clinical applicability

**Figure 7:**
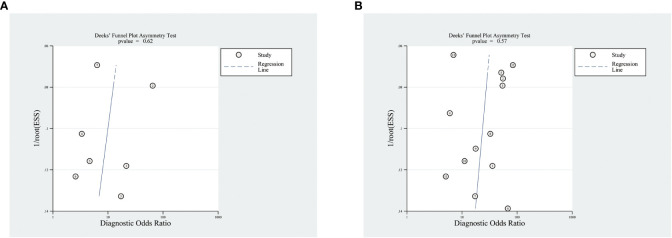
Funnel diagram of SMI and CDFI. Panel **(A)** is the funnel diagram of CDFI; panel **(B)** is the funnel diagram of SMI.

**Figure 8:**
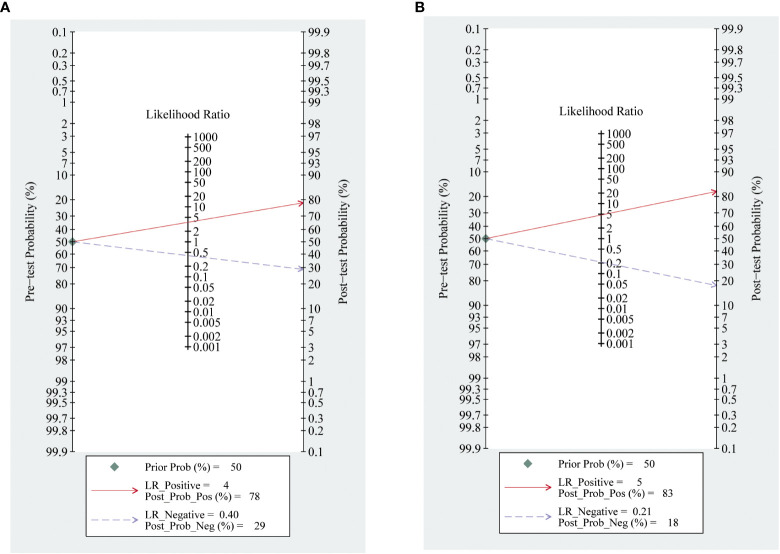
Fagan diagram of SMI and CDFI. Panel **(A)** is Fagan diagram of CDFI; Panel **(B)** is the Fagan diagram of SMI.

The meta-analysis of the value of SMI and CDFI showed no significant asymmetry (P>0.05), that is, there was no significant publication bias (**Figure 7**). At the same time, it can be seen from the Fagan diagram that the post-test probability of SMI and CDFI is 83% and 78% respectively, which is 50% higher than the pre-test probability. The combined negative likelihood ratio of SMI and CDFI in the diagnosis of malignant thyroid nodules is more than 0.1, and the combined positive likelihood ratio is less than 10, indicating that both methods are effective in the diagnosis of malignant thyroid nodules (**Figure 8**).

## 4 Discussion

At present, the effective non-invasive diagnosis and differential diagnosis of benign and malignant thyroid nodules is an urgent problem to be solved. Although CUS can display the size, echo, boundary, calcification and blood flow of nodules, it has certain limitations in displaying the blood flow of lesions. SMI has unique advantages in displaying blood vessels. It can better display low-velocity blood flow without the use of contrast media. Diseases closely related to angiogenesis can be diagnosed at a relatively early stage through SMI. It is of great significance to evaluate the grading and treatment effect. At the same time, SMI can avoid adverse reactions related to CEUS contrast agents. However, SMI is at the stage of clinical exploration and has not been widely used. Specific clinical standards have not been established for the diagnosis of some diseases. There are still many disputes about the differential diagnosis of benign and malignant tumors. Therefore, this study uses the meta-analysis method to systematically evaluate and compare the value of CUS combined with SMI and CDFI in predicting thyroid malignant nodules, to provide an evidence-based basis for the differential diagnosis of thyroid malignant nodules.

Neovascularization and the growth of irregular vascular structures are the prominent characteristics of malignant tumors (34). In contrast-enhanced ultrasound (CEUS) examination, thyroid cancer below 1 cm usually shows low enhancement (35, 36). However, some researchers found that with the enlargement of the nodule, the blood flow inside the nodule is gradually enriched, 1-2 cm thyroid cancer often shows moderate to low enhancement, and>2 cm thyroid cancer often shows high enhancement, at the same time, benign nodules mainly have peripheral and mixed blood flow distribution patterns, and the central blood flow distribution pattern is a significant sign of malignant nodules (37–40). Frates described the CDFI features that may help to distinguish benign and malignant nodules (8). Cappelli evaluated the number of blood vessels in thyroid benign and malignant nodules and showed that thyroid malignant nodules had rich blood flow signals, which confirmed that the number of blood vessels was an important parameter for distinguishing thyroid benign and malignant nodules (33). Kong showed that the specificity of SMI in predicting intra-nodular vessels was 91.2%, and intra-nodular vessels were an effective feature in predicting malignant tumors (28). Zhao performed SMI and CEUS examination on 102 thyroid nodules, the diagnostic value of SMI-BF of the periphery is comparable to CEUS hypoen-enhancement to differentiate thyroid cancer, analysis of SMI-BF as diagnostic of thyroid carcinoma specifically at the nodule periphery indicated rates of 82.2%, 79.3%, and 81.3% for sensitivity, specificity, and accuracy, the corresponding rates for CEUS hypoen-enhancement were 80.8%, 86.2%, and 82.3%, respectively (29). Compared with SMI, CEUS can display more blood flow information inside the nodule, but the price is relatively expensive. The detection rate of CDFI for low-speed blood flow is low, especially for low-speed blood flow<1cm/s, so the evaluation of small and low-speed blood flow has limitations. When the penetrating vessels were used as the characteristics of malignant nodules, the results showed that the sensitivity, specificity, accuracy, and other results of SMI diagnosis of thyroid nodules with a diameter of less than 1 cm were not statistically significant with those of CEUS, which showed that SMI and CEUS had similar diagnostic capabilities in the differential diagnosis of thyroid nodules.

This study used the diagnostic experimental evaluation tool QUADAS-2 to evaluate the quality of the included literature. The results showed that the overall quality of the included research was high and the risk of bias was low. However, the risk of bias is high in the selection of study subjects, which may be since some studies only include patients suspected of thyroid cancer, which leads to bias, and some studies do not report the mass spectrum composition of the study population, which leads to the fact that the included population cannot represent the clinically accepted population, which has an impact on the accuracy of the test. Therefore, the criteria for the inclusion of cases should be clarified and the process of inclusion of cases should be described in detail to improve the possibility of clinical application of diagnostic tests. In addition, more than half of the documents included in this study did not report the time interval of imaging examination and diagnostic criteria, which may also lead to the bias of results. The heterogeneity included in the study is large, but the heterogeneity test indicates that there is no threshold effect, and the heterogeneity may be mainly caused by the non-threshold effect. This may be caused by the characteristics of the patients included in each study (nodule classification grade, nodule diameter size), the technical level of the operator, the type of research design, and the use of different diagnostic criteria. Meta-regression analysis shows that the relationship between the heterogeneity sources of CDFI and SMI and the covariates is not statistically significant, and it is speculated that the operator dependence of the relevant measurement will also bring some deviation. The heterogeneity of this study is not caused by the threshold effect, so a random model is used for summary analysis. In addition, this study is a diagnostic meta-analysis, so there is inevitable clinical heterogeneity.

To evaluate the ability of SMI and CDFI in the differential diagnosis of thyroid nodules, a total of 13 articles were included in this meta-analysis, including 1712 nodules. The combined sensitivity of SMI in the diagnosis of thyroid malignant nodules was 80%, the specificity was 79%, and the misdiagnosis rate was 20%, which was relatively high. The positive likelihood ratio was 4.37, the negative likelihood ratio was 0.23, the diagnostic odds ratio was 22.29, and the area under the SROC curve (AUC) was 0.8944. The combined sensitivity and specificity of CDFI in the diagnosis of thyroid malignant nodules are 62% and 81%, the positive likelihood ratio was 3.33, the negative likelihood ratio was 0.41, the diagnostic odds ratio was 8.93, and the area under the SROC curve (AUC) was 0.8498. SROC curve is a comprehensive index that directly observes the accuracy of diagnostic tests and reflects the sensitivity and specificity of diagnostic tests and target disease. AUC value is an important test accuracy index. The closer it is to 1, the better the diagnostic efficiency of this diagnostic method is. The results of this meta-analysis showed that the combined sensitivity, and ratio of SMI were higher than those of CDFI, and the area under the SROC curve was also larger than that of CDFI, indicating that SMI was more effective than CDFI in diagnosis.

This study has limitations. First, most of the included documents are from China, which may lead to potential regional bias. Second, not all the literature is prospective, and retrospective literature may improve the accuracy of diagnosis. Thirdly, due to the insufficient number of documents with nodule diameter<1cm included in the study, the ability of SMI to differentiate and diagnose thyroid micro-nodules could not be evaluated separately, and more research reports on SMI to differentiate and diagnose thyroid micro-nodules are needed. Fourth, the two methods of examination and diagnosis are not quantified, and there is no unified diagnostic standard. Fifth, although diagnostic meta-analysis has inevitable clinical heterogeneity, reducing heterogeneity is conducive to improving the accuracy of meta-analysis. Because there are relatively few reports on the diagnostic performance of SMI in differentiating benign and malignant thyroid nodules, a large-scale multi-center study is still needed.

In conclusion, SMI is superior to CDFI in the diagnosis of thyroid malignant nodules, and SMI technology can reflect the distribution of blood flow, avoid the overflow of blood flow signals, and can sensitively detect low-velocity blood flow and micro vessels to make up for the deficiency of CDFI, with good clinical application value.

## Data availability statement

The original contributions presented in the study are included in the article/supplementary material. Further inquiries can be directed to the corresponding author.

## Author contributions

LJ and DZ: Software, Writing - original draft. Y-NC: Writing – review and editing. X-JY: Visualization, Investigation. M-FP: Supervision. LL: Conceptualization, Methodology, Supervision. All authors contributed to the article and approved the submitted version.
